# Supply Management 2.0: A Policy Assessment and a Possible Roadmap for the Canadian Dairy Sector

**DOI:** 10.3390/foods10050964

**Published:** 2021-04-28

**Authors:** Sylvain Charlebois, Eric Bowdridge, Jean-Luc Lemieux, Simon Somogyi, Janet Music

**Affiliations:** 1 Agri-Food Analytics Lab, Dalhousie University, Halifax, NS B3H 4J1, Canada; er542386@dal.ca (E.B.); jeanlucwlemieux@gmail.com (J.-L.L.); janet.music@dal.ca (J.M.); 2 Arrell Food Institute, University of Guelph, Guelph, ON B2T N1G, Canada; ssomogyi@uoguelph.ca

**Keywords:** supply management, Canadian dairy, overcapitalization, public opinion, public policy, survey

## Abstract

Many believe the current Canadian Dairy supply management system is outdated. Examining a recent consumer survey suggests consumers, especially among the younger generations, have mixed feelings about how the Canadian dairy industry is good for the environment or whether animals in the sector are humanely treated. The general Canadian public strongly supports financial stability for farmers, though is not fully educated about how supply management works. Issues regarding the centralization and amalgamation of the industry, making many regions underserved; recent milk dumping due to a strong shift in demand caused by COVID-19; and the popularity of dairy alternatives, show that the dairy sector in Canada is ill-prepared for major change. Dairy farmers are receiving compensation for trade deals recently ratified by the federal government, creating a precedent that will lead to an overcapitalized industry. The aim of this paper is to review the industry’s current state and suggest a roadmap for a more prosperous future.

## 1. Introduction

Supply management continues to be a controversial subject in the Canadian dairy industry. As many countries have phased out their supply management programs, like Australia, the United Kingdom, Korea, and the European Union [1], the recent Trudeau government compensation of CAD (Canadian) 1.75 billion to dairy farmers for trade deal losses [2] created a precedent, experts have debated the purpose and importance of supply management in Canada. The main purpose of supply management is to produce agricultural commodities consumed domestically. It is achieved by implementing production quotas and setting high tariffs applied on imported goods, considered as main pillars of the system. Supply management affects poultry, eggs, and dairy, but the latter sector represents about 80% of cash receipts govern under supply management [3].

Supply management is seen as a controversial topic, and the system itself is facing mounting challenges due to newly ratified trade deals and changing market trends. The aim of this study to appreciate the dairy industry’s current state while looking at the future for a potential strategic roadmap for the industry. A concise literature review on the Canadian dairy industry was undertaken. This contextual literature review covers the founding of supply management in Canada, historical influencers, modern influencers, assessments of the dairy industry, impact of supply management on dairy farmers and the Canadian public, and current factors pressuring dairy farmers. To fully comprehend the situation and be able to make meaningful policy change, understanding supply management’s history as well as its modern context is critical. This includes critically analyzing the formation, components, influencers, and practices. The literature review uses a combination of academic papers, grey literature, journals, news articles, and independent reviews, along with relevant policies, acts, and government statements. There has also been consideration for non-government organizations and dairy farmers, regarding their role in the system. Review of their prepared documents have been utilized for determining their current and historic roles in the supply management of dairy. Using this literature review, key stakeholders and drivers in the industry are identified.

### 1.1. Context

In Canada, the price of dairy products in Canada at farmgate is based on production quotas set by the Canadian Milk Supply Management Committee (CMSMC) by assessing the demand for milk and establishing a fair price for these quotas based on the costs of input for farmers [4]. The CMSMC is chaired by the Canadian Dairy Commission (CDC), a crown corporation which works in conjunction with provincial milk marketing boards to decide which total quota should be allotted, based on consumer demand. The quota is then distributed amongst the provincial milk marketing boards to be allocated to farmers in each province. The CDC was created with the purpose of ensuring that Canadian dairy farmers receive fair prices for their output, and so processors could depend on stable input for their products, to limit shortages and surpluses. The CDC is also responsible for determining the farm gate price [5]. The farm gate milk price is the minimum price of industrial milk and is based on a national pricing formula that considers dairy farmers’ costs of production and the consumer price index [6]. On 1 February 2020, under the normal pricing structure, the farmgate price increased by 1.93% [7], this translates to an approximate increase of CAD 1.46/hL [8]. That percentage will fluctuate from year to year. Due to COVID-19, the CDC has conducted consultations to determine the price adjustments of milk instead of using the standard pricing formula. Through these consultations, the CDC increased farm gate milk price by CAD 1.46/hL, or 2.0%, on 1 February 2021 [9,10].

Within the past four years, Canada has ratified three new trade agreements: The Comprehensive Economic and Trade Agreement (CETA), The Comprehensive and Progressive Agreement for Trans-Pacific Partnership (CPTPP), and The Canada–USA–Mexico Agreement (CUSMA). CUSMA was entered into force on 1 July 2020. All three trade deals have created breeches in the country’s tight supply management policy, which allows a significant amount of imported dairy products to enter the Canadian market, tariff-free. Expert examining changes from the North American Free Trade Agreement (NAFTA) to CUSMA have highlighted factors like the removal of milk price Class 6/7 and new limits on exports of skim milk powder, milk protein concentrate, and infant formula. CUSMA’s full effects have not yet been realized and anticipated impacts remain highly speculative. It is considered a source of worry for the Canadian Dairy industry [11], especially with recent US accusations that Canada is “unfairly limiting the ability of American dairy producers to sell their products north of the boarder.” These accusations specifically attack Canada’s supply management system stating that the Government of Canada (GoC) “give[s] market access to competitors with little incentive to take advantage [of it.]” [12].

### 1.2. More Trades

Under these new agreements, Canada’s supply managed dairy industry finds itself in a precarious position. The CPTPP has cost the industry 3.25% of its market (CPTPP, 2020), CETA consumes an additional 1.3% of the market [13], and CUSMA recently gave an additional 0.5% access to American dairy farmers (who already receive 3% of Canada’s market) [14]. The combined effect of these free trade agreements will harm the industry in the short-term by 8.05% of the Canadian domestic market, limiting domestic production quotas, and taking revenue away from Canadian dairy farms. The Dairy Farmers of Canada (DFC) estimates the annual potential loss to farmers from these new trade agreements to be 8.4% of total production [15]. In recent statements, DFC claims that these trade concessions will account for 18% of domestic milk coming from outsourced dairy farming [16], and the introduction of more foreign products will generate competition for domestic products, leading consumers to drink less Canadian milk [17]. In response to the concern of dairy farmers and proponents of supply management, the Liberal government announced on 16 August 2019 that it will be compensating the industry with CAD 1.75 billion over eight years for losses to farmers from the CPTPP and CETA, with future subsidies planned for CUSMA [18]. More compensations are expected for CUSMA [19].

**Figure 1:**
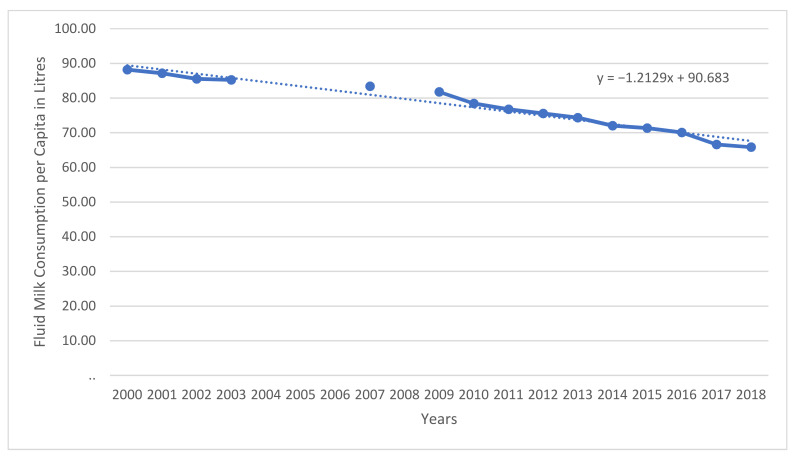
Annual Total Fluid Milk Consumption in Liters per Capita Canada (2008 to 2018 where Data Available). Source: Statistics Canada; Compiled by Agriculture and Agri-Food Canada, Animal Industry Division, Market Information Section.

As a national lobby for farmers, DFC has played a major role in organizing farmers and dairy advocates into action to earn these protections, launching the My Canadian Milk advocacy movement [20]. However, the capabilities of this powerful lobby may be hampered in the future by what is currently happening to the industry. As of 2018, the rate of fluid milk consumption in Canada has been falling consistently for the past 20 years [21], as we can see from Figure 1. Facing major budget cuts, the DFC has had to adapt, launching a new strategic advertising campaign aimed at increasing dairy product consumption amongst millennial parents to attract a new generation of milk drinkers with the “Milk. It’s in the Stuff You Love” campaign [22].

### 1.3. Change in Consumption

**Figure 2:**
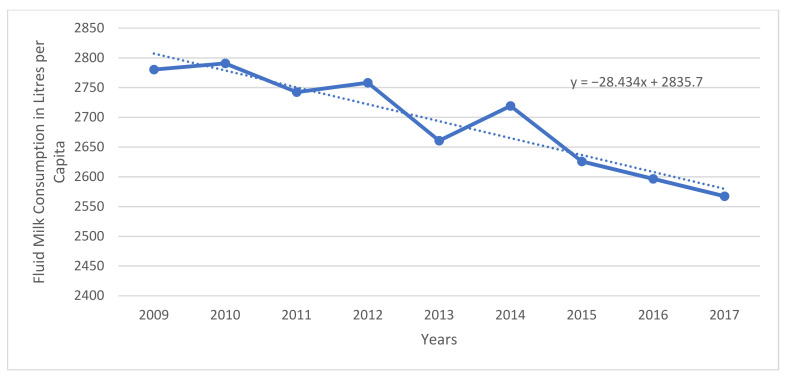
Global Fluid Milk Consumption in Liters per Capita (2009 to 2017 for Countries in which Data for all Years is Available). Source: Statistics Canada; Compiled by Agriculture and Agri-Food Canada, Animal Industry Division, Market Information Section.

**Figure 3:**
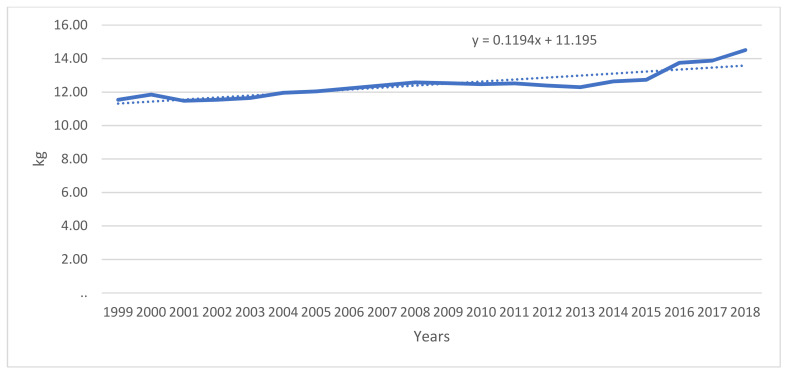
Total Cheese Sales in kg per Capita (Canada, 1999 to 2018). Source: Statistics Canada, n.d.

Fluid milk consumption is not only decreasing in Canada, but global consumption is trending downward as well, as can be seen in Figure 2. Asia is considered the only real emerging market [23]. DFC, among others, has recognized this trend, and has begun focusing its marketing on non-milk dairy products, recognizing that there has been a small trend of growth in the consumption of these products [24], which can be seen in Figure 3 for cheese.

The dairy has also often faced public criticism for not listening to consumers. In 2021, consumers noticed a change in butter texture. Soon after, it was reported palm fats were used as dietary supplements for lactating cows, to produce more butterfat. Most Canadians were not aware of such practice. The episode is also known as “ButterGate” in Canada. Consumers realized that not only dairy product quality has been compromised in recent, the use of palm oil in dairy production came as a complete shock for many Canadians, which affected the industry’s image. While Canadian dairy farmers promote the sustainable and local nature of supply management, palm oil is imported and known to be link to unsustainable and destructive production practices in Asia. Supply management essentially prevents farmers from reading cues from the public. The use of palm oil as a supplement is legal in Canada since 1983, but the market has changed significantly in four decades.

### 1.4. Dismantling Supply Management

Inspired by the Keynesian school of economic thought, supply management in the Canadian dairy industry is an insightful example of the costs and benefits of protectionist economic policies. Because of supply management, major dairy producers have thrived in Canada while avoiding the pressures of the global economy, and consumers have avoided drastic price fluctuations in dairy products. For the most part, supply management has ensured processing plants have had a consistent supply of safe, high-quality milk to make products, and has ensured that milk is not over-produced, spoiling before reaching the shelves of supermarkets. The cost of these benefits has been high dairy prices in Canada, low levels of innovation in the industry, and an unfavorable starting point for trade negotiations [25]. With the pressure of COVID-19 on the dairy industry, supply management quotas have led to the dumping of 5 million liters of milk a week in Ontario as of 6 August 2020, who produces roughly 3 billion liters of milk a year [26]. It is unknown when/if the milk dumping has stopped. Other provincial marketing boards in Quebec, Nova Scotia, New Brunswick, and Newfoundland, and Labrador have also asked dairy farmers to discard significant amounts of their yearly milk production [27]. DFO have stated that the milk waste is due to reduced demand because of the closure of restaurants, hotels, schools, and other bulk buyers. They claim that the dumping of fresh milk will balance out supply and allow prices to remain stable [28]. Once artificially inseminated, cows cannot stop producing milk [29], and the strategic reserve managed by the CDC does not have the capacity to handled massive sudden excesses of milk and dairy products. With the pricing formula managed by the CDC, farmers are compensated for discarded milk over time [30]. Farmers are essentially incentivized to waste milk since penalties would apply, should off-quota milk be brought to market. Additionally, they claim the change in demand due to COVID-19 was an “[…] unpredictable market [fluctuation]” meaning there was no emergency planning for reduced demand [31].

According to the Conference Board of Canada report, “Reforming Dairy Supply Management: The Case for Growth”, supply management in the dairy industry should be dismantled, and trade should become fully liberalized, but dismantling supply management is not a viable solution for Canada. If trade were liberalized tomorrow, then American milk would likely flood the Canadian market. Canada’s farmers would not be able to compete with the price of American milk, and eventually the entire Canadian dairy industry would be dependent on imported milk [32]. Seasons in Canada make it more expensive to produce milk, especially during the Winter months. The pure free-market economic model tells us that the Canadian dairy industry cannot currently compete with the marginal production costs of dairy producers in other countries. Therefore, it would be more efficient for Canada to dismantle supply management, halt all subsidies for the dairy industry, and allow consumers to access cheaper international dairy [33]. While free-market economics may produce an efficient outcome, it does not necessarily create an optimal situation when we consider the livelihood of dairy farmers, domestic food autonomy concerns, our rural economy, and our values as Canadians.

A basic strategy for dismantling supply management would likely be for Canada to follow the Australian example, as has been discussed by Findlay et al., [34], for instance. The plan would go as follows: remove tariffs on dairy products, import cheaper dairy, put a levy on dairy products for consumers, generate money (from the levy) to purchase milk quotas back from farmers, and set a 10-year course for purchasing all dairy quotas from farmers before fully entering a free-market system. The idea for Canada to adopt a model like Australia’s for dismantling supply management is not new. Policy analysts have been recommending Canada follow suit since at least 2003 [35]. While this plan would reduce costs, it would inevitably lead Canada into a similar situation as Australia currently finds itself in. In Australia (due in part to massive drought, heat, and high animal feed costs), the market has been devastated by the removal of supply management [36]. As reported by Australia’s national dairy service body, Dairy Australia, since deregulating the market, farmers have had to struggle to compete with international milk prices. “At an average of approximately USD 42 cents per liter, Australian dairy farmers receive a low price by world standards, and therefore have to run very efficient production systems.” [37]. In 1980, there were 22,000 dairy farmers in Australia; however, today there are fewer than 6000, and milk production was forecasted to fall by as much as 9% in 2019, according to an article from ABC News called “Australia’s dairy farmers issue warning as mass exodus continues.” [38]. Similar situations are occurring in other markets with free-market dairy policies, such as in New Zealand, the UK, and the US [39].

Furthermore, proponents of dismantling supply management will demonstrate that Canadians pay more for milk than Americans; however, when we consider the direct and indirect subsidies which American dairy farmers receive, we may not be as convinced that the price is so different. According to Alberta Milk, “Americans paid $4 billion in dairy subsidies in 2009, and about 31 cents per liter, in addition to retail prices” [40]. According to an article from RealAgriculture.com, “Support, in its various forms, equaled 73 percent of U.S. dairy farmers’ market returns in 2015” [41]. In fact, American dairy farmers in Wisconsin have recently been discussing asking the federal government to become more involved in the industry, as farmers are currently struggling. Some are even considering proposing a system like Canada’s current supply management system [42].

Finally, the loss to Canada’s economy from protecting the supply management system cannot be denied. Tariffs create deadweight economic loss according to a study entitled “The Annual Loss to Canadian Society Caused by Supply Management of the Dairy Industry” by Kelly Davey. Supply management of the Canadian dairy industry results in a roughly CAD 123 million in deadweight loss to the society annually [43]. Davey’s calculations used data from the 1997–1998 data year; more recent calculations are yet unknown.

### 1.5. History of Supply Management

**Figure 4:**
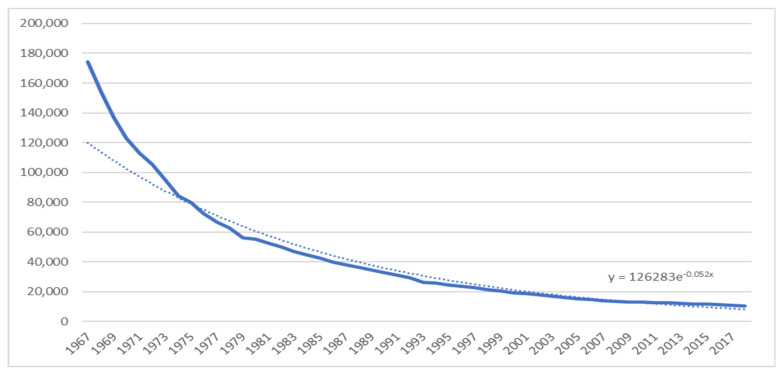
Number of Dairy Farms in Canada (1967 to 2018). Source: Canadian Dairy Information Center, 2019.

Historically, supply management made sense; agricultural products are subject to inelastic demand, and prices can fluctuate drastically based on small changes to supply [44]. By ensuring prices remain stable through a quota system, farmers can maintain their herds regardless of disturbances in consumer demand, and consumers can depend upon stable dairy prices regardless of disturbances in supply. At the time of the creation of supply management, farms were much smaller, and it took many more farms to produce the amount of milk which fewer farms produce today. In fact, in 2018, there were only 10,679 dairy farms [45] while in 1971 at the outset of supply management there were 145,000 dairy farms [46]. The farms have been exponentially decreasing as represented by Figure 4.

**Figure 5:**
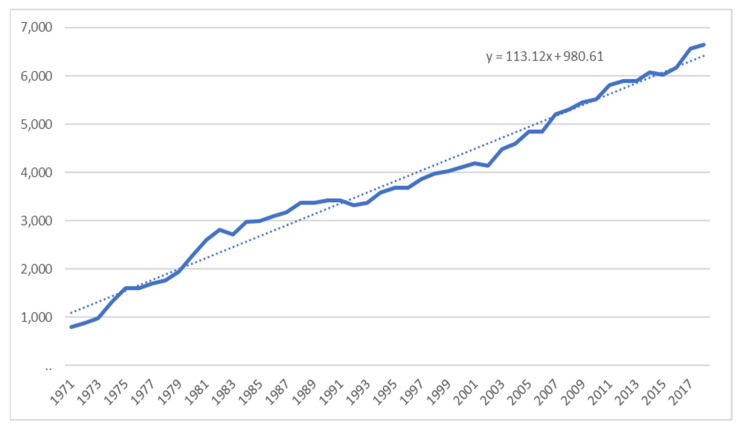
Canadian Farm Cash Receipts (×CAD 1,000,000) from Dairying in Canada 1971 to 2018. Source: Canadian Dairy Information Center, 2019.

While there are fewer farms, these farms have much higher revenues than those of 50 years ago [47], as we can see from Figure 5, which shows the growth in farm cash receipts from dairying in Canada from 1971 to 2018, in millions of dollars. When supply management was created, dairy farmers had limited means to communicate and gather market intelligence to fully access market conditions. Today, most farmers are highly educated and can run their operations with start-of-the-art equipment [48].

**Figure 6:**
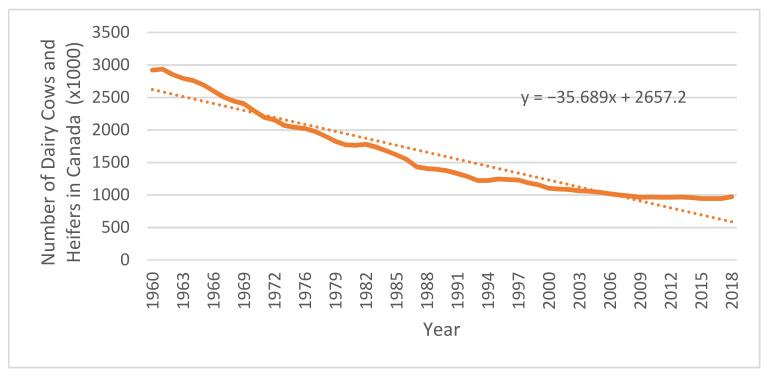
Number of Dairy Cows and Heifers in Canada (1960 to 2018). Source: Canadian Dairy Information Center, 2019.

Since the advent of supply management, genetics and animal science have also changed drastically, allowing for a much higher yield of milk with fewer cows [49]. In 1971, total milk production was 76,321,710 hectoliters; by 2012 it had hardly changed at 79,801,292 hectoliters [50]. However, this level of production was achieved with far fewer farms and fewer total cows producing the milk, as shown in Figure 6. On the other hand, these statistics have garnered some criticism by animal rights groups, stating that dairy cows are being denaturalized and inhumanely exploited [51]. Technology developments are also set to impact the Canadian Dairy Sector. Advances in precision fermentation will allow for the production of milk proteins in an industrial setting, providing milk and milk fats identical to those made by a cow. Some report suggests this technology will be in commercial operation within the next 15 years [52].

While supply management has offered farmers stable prices for dairy, Canadian processors must still compete for the domestic market. Within all markets with a dairy industry, competition has led to economies of scale, including in Canada, regardless of the current supply management system. To reduce input costs, farmers have increased farm size and amalgamated production. This said, Canada has not taken as much advantage of scaling as the rest of the world and would fail to compete internationally [53]. The decision of the industry to move toward economies of scale to reduce production costs has resulted in a disproportionate number of farms grouping in some areas of Canada: 42% of the total MSQ (Market Sharing Quota) for dairy is in Quebec, and 32% in Ontario. Over 74% of all dairy farms are in two provinces where 61% of our country’s population resides [54]. This is problematic as having large farms located in only one or two provinces may not be the most sustainable or beneficial solution for Canada’s industry going forward. Figure 4 shows the number of dairy farms and Figure 6 shows the number of dairy cows in Canada, showing how the number of small farms in Canada has declined dramatically since the establishment of supply management. When we look at the trend line of the Number of Dairy Farms in Canada by Year, we find the best fit to be an exponential formula of the form: y = 126,283e^−0.052x^. We can extrapolate from this; that is, if the estimate used is correct, in ten years’ time we can expect the number of farms to have halved again, to roughly 5500 farms by the year 2030. This prediction is consistent with other observations made [55]. Maintaining domestic production capacity is critical to the success of the sector and to our nation’s food security, but it remains unclear how the industry can remain strong with our current policy framework.

While consumers in Canada can depend on a somewhat stable cost of milk when planning their monthly grocery budget, they can also expect to spend much more at the grocery store than their American neighbors. From 1997 to 2011, the price of milk in eastern Canada averaged CAD 63.05/hL, while the U.S. Midwest price averaged CAD 39.42/hL and New York/New Jersey averaged CAD 44.31/hL [56]. More recent estimates come from an article published on 2 December 2019 in the Toronto Sun, which was based on a study that confirms that Canadians pay almost 30 cents more per liter of milk [57]. However, the evidence which suggest that milk and dairy products would be more affordable if supply management ends is weak, at best. The laws of supply and demand in a vast country like Canada set new market conditions which are difficult to predict. Given the cost of distribution to cover the Canadian market, depending on where products are coming from, Canadians may very well pay more for dairy products, once supply management ends [58]. This said, creating a free-market system, introducing large quantities of highly subsidized American milk, as aforementioned by Alberta Milk, may not have the positive results many expect.

Canada’s ability to negotiate trade deals has arguably been undermined by high tariffs imposed on dairy imports into Canada. Canada will seek to maintain its domestic monopoly on dairy products, restricting competition with high tariff rates. Other countries will also have similar non-negotiable issues. Tariffs on dairy products coming into Canada are generally more than 200% [59]. Canada’s dairy industry has been referred to as a “legal cartel,” and some authors suggest dismantling supply management altogether in favor of consumers [60], while others suggest doubling down on supply management and restricting further market access for dairy products [61,62]. In recent years, the issue has become quite political, as witnessed in full force during the lead-up to the 2019 Federal election.

### 1.6. Present Study

With recent compensations, under our current regime, the risk of creating an overcapitalized industry is high and could cause the number of dairy farms in Canada drop significantly is significant [63]. It is critical that new policy counters centralization and allows the dairy industry to succeed equally in all regions. Right now, most of the industry is in Central Canada which compromises the producers’ ability to fully occupy the vast Canadian market. This said, DFC still argues that the supply management system has improved over the years [64]. New policy reforms for the industry will need to be based on the principles of transparency, accountability, and economic viability for the system. There is need for assessing how the system is assisting and helping (or hurting) the dairy industry, with consideration of both the domestic and global markets. An assessment with considerations for the history and context of supply management, would give perspective to the competitiveness and health of the system. These components are crucial for the proposition of new policy.

The current supply management system could be optimized and improved, though the industry continues to struggle [65]. For years, experts have either defended the current regime [66] or have chastised it and called for it to be abolished [67,68]. However, in our literature search there was no data on where the Canadian public stands regarding this debate, meaning there is also need for measurable public opinion and public understanding related to the system, which must impact policy for the future. The research conducted intends to analyze the knowledge of the Canadian public regarding the functions of supply management. It is important for policy makers to not only consider the voices of academics but also those of the Canadian public, and how the informed (or uninformed) opinions of the Canadian public on supply management result in potential overcapitalization of the industry, which is what this paper aims to answer. Our hypothesis is that the wants of Canadians will ground the current actions being taken by the GoC regarding supply management. The conducted national survey by the Agri-Food Analytics Lab (AAL) at Dalhousie University tested the hypothesis by clearly identify the knowledge base of the Canadian public on supply management, their perception of the importance of the dairy industry, their financial flexibility, and their opinions on if they believe the Canadian dairy industry is good for the environment or whether animals in the sector are humanely treated.

## 2. Methods

In December 2019, a national survey was conducted by the AAL at Dalhousie University, which aimed to show the underpinnings of the Canadian public’s knowledge of supply management. This perspective on supply management is vital for understanding the impacts of new dairy policy in Canada, though such a study had been missing up to this point. Survey questions were created to determine the actual knowledge of the Canadian public regarding supply management, their opinions on the Canadian dairy industry including if they believe the industry is good for the environment or whether animals in the sector are humanely treated, and their consumption behaviors and price elasticity. Survey design was based on instruments used in past studies [69,70], and new questions were added to assess perceptions related to holistic issues like animal welfare and the environment. The instrument was validated by key experts in the field of market research. Internal (Cronbach’s alpha) and external consistencies were also verified. The survey relied on Likert Scale scoring with the following terms:

“Strongly agree, somewhat agree, neither agree nor disagree, somewhat disagree, and strongly disagree.”

Considerations for inconsistent answers resulted in the use of common practices like reverse scoring. There was also opportunity for comments on most topics. This survey had 1143 randomly selected participants with proportionate representation from each region of Canada. The margin of error in the survey is 2.9% or 19 times out of 20.

## 3. Results of the Agri-Food Analytics Lab National Survey

Seeking a fuller picture, an evaluation of the hypothesis was completed by compiling a survey of Canadian opinions on the dairy industry and supply management by the Agri-Food Analytics Lab at Dalhousie University. Noting that consumers are at the foundation of the entire dairy industry, the results are informational. Currently, 33% of Canadians are entirely uninformed about supply management and one respondent replied:

“There needs to be more education about it as I know very little about it (mostly hear advocates praise it and others whine about it).”

Many others expressed similar concerns. Additionally, 44% of Canadians believe the beef industry is protected by supply management. The survey suggests that there is a lack of understanding of the functions of supply management in the dairy industry and supply management industries. This lack of understanding is not new as many Canadians have not understood the essence of the regime [71]. Further, the current dairy pricing mechanisms are shrouded in mystery, with only 9.21% of Canadians strongly agreeing with the statement: *“I understand how the price for milk on the farm and at retail are determined in Canada.”* The survey showed that most Canadians know very little about supply management. Survey results suggests that only 17.2% of Canadians feel that they can strongly agree with the statement: *“I am familiar with supply management in the dairy industry.”* Furthermore, many Canadians believe that the salmon, wheat, maple syrup, and beef industries are also regulated by supply management. In fact, only dairy, poultry (chicken and turkey), and eggs are federally supply managed [72,73].

Based on the national survey, 53% of respondents strongly agree that dairy farming is an important industry in Canada, while 66% somewhat agreed or strongly agreed to the statement: *“Dairy farmers in our country are an important part of what it means to be a Canadian.”* 62% responded that they strongly agree that it is important to support Canadian dairy farms by buying Canadian dairy products, and 60% somewhat agreed or strongly agreed that supply management is good for the Canadian economy. A total of 62.8% of respondents somewhat agreed or strongly agreed that the government should protect supply management in the dairy industry. These results show that Canadians care about dairy farmers and want them to succeed. The majority are even willing to protect them at their own expense. Regarding the statement, *“If dairy products were more expensive, I would still purchase them if it means that we can keep dairy farms in Canada,”*; 66% of respondents somewhat agreed or strongly agreed. 50% of Canadians even agree somewhat or strongly that it is important to support Canadian dairy farms by providing direct subsidies to them. These results suggest that half of Canadians are even willing to spend more for dairy products to protect the domestic industry. With this foundation established, the data shows that Canadians do not want to see the end of the domestic dairy industry. Dairy farming is an important part of what it means to be a Canadian, and most respondents (85.5%) somewhat agreed or strongly agreed that it is important to support Canadian dairy farms by purchasing Canadian dairy products. Canadians want Canadian dairy products. The demand exists, and the support for the system and its producers is evident.

When we look to consumers for direction, we find evidence of a few things. First, consumers not only prefer food grown in Canada, but they would also prefer to eat food grown within their own province. A total of 86.9% either somewhat agree, or strongly agree with the statement: *“I prefer to eat food produced within my province or region if possible.”* Most respondents want food that is sustainably sourced and are willing to pay more for food which ensures animals are treated humanely. Furthermore, 55% believe that smaller farms treat animals more humanely than larger farms. Additionally, 82.5% of respondents either somewhat agreed or strongly agreed with the statement: *“I believe it should be a priority to protect jobs in rural Canada.”* However, another indication for the importance of maintaining dairy farming, and processing, in all regions of the country.

**Figure 7:**
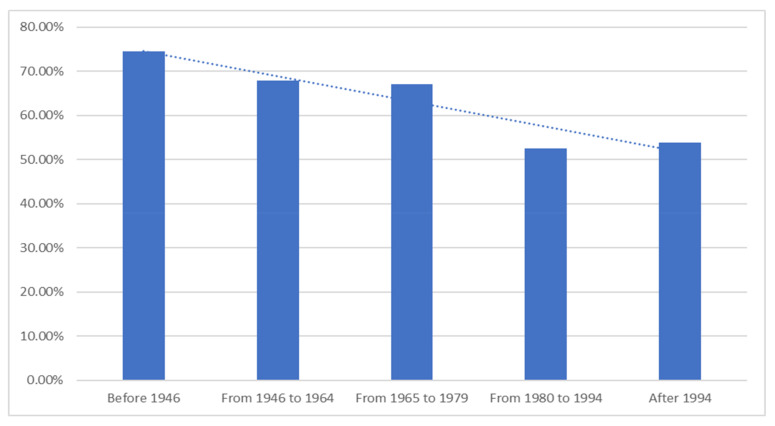
I believe it is important to support Canadian dairy farms by buying Canadian dairy products.

While Canadians may not understand how the dairy industry operates, or how supply management works, they overwhelmingly responded in favor of supporting the industry. In total, 87.3% of Canadians either somewhat or strongly agreed when presented with the statement: *“I believe dairy farming is an important industry in Canada”*. However, when we look at the responses divided by age group, we find a clear pattern emerging: the support for the dairy industry declines with each successive generation. We find that individuals born before 1946 are more likely to agree or strongly agree with the statement (98.4%) than those born between 1965 and 1979 (87%), and much more than those born after 1994 (80%). This trend can be seen in Figure 7. Canadian support for the dairy industry is decreasing with each generation. Similar findings were found for the question: *“Dairy farmers in our country are an important part of what it means to be a Canadian,”* where agreement fell from 82.5% in the eldest generation to 56.3% in the youngest generation. One Canadian even replied to our survey with the following statement:

“[…], Dairy is an outdated and unnecessary food—Canadian dairy farmers should get with the times and switch to agriculture. The future is vegan and even the Canada Food Guide supports this reality.”

Furthermore, we found that when we asked Canadians if they agreed with the statement: *“I believe it is important to support Canadian dairy farms by buying Canadian dairy products,”* overwhelmingly they agreed (85.6%). Similarly, we found that Canadians would also prefer to eat dairy products produced locally than foreign products, regardless of whether they are more expensive (62.8% disagreed with the statement: *“I prefer to eat food imported from foreign countries if it is less expensive than Canadian food.”*). However, in both cases, the results varied quite significantly by age: 74.6% of individuals born before 1946 strongly agreed, while only 52.6% of those born between 1980 and 1994 strongly agreed. Similar results were then found for the statement: *“I prefer to eat food imported from foreign countries if it is less expensive than Canadian food.”* The number of people who strongly disagreed with the statement fell from 30.3 to 16.3% across generations. These differences in generational perspectives are, perhaps, the most interesting results of this analysis. Another generational difference which could potentially explain this gap is the result that younger people were more likely to claim they understand how the industry works than older generations. A total of 45% of people born after 1994 agree with the statement: *“I understand how the price for milk on the farm and at retail are determined in Canada,”* while only 40.5% of those born between 1946 and 1964 agreed. This could imply that knowing more about the current supply management system is in fact encouraging younger generations, more than their senior counterparts, to feel that the dairy industry is less important. Or, this could be a false correlation, and the results could be explained differently. As a side note, women were also less likely than men to agree (37.1% of women compared to 44.1% of men agreed).

When it comes to awareness about supply management, as stated above, people appear to be less than adequately informed. However, for those who do know, or at least have a vague understanding about it, most (60.3%) agreed with the statement: *“I believe supply management is good for the Canadian economy,”* with women (64.3%) agreeing more often than men (56.1%). Those who agreed that supply management was good for the economy were mainly centered in Quebec, Ontario, and Atlantic Canada (65.7%, 63.6%, and 60.2% respectively). When we continued this line of reasoning with the statement: *“I believe the government should protect supply management in the dairy industry,”* we found similar results, as can be seen in Table 1. Interestingly, the crosstab pertaining to type of locale (i.e., small town, suburban, or urban) was significant for this question, with results showing that people in small towns were less likely to agree (57.5%) than those located in urban cores (65%).

Canadians even support direct subsidies for the dairy industry, with 51.6% agreeing with the statement: *“I believe it is important to support Canadian dairy farms by providing direct subsidies to them,”* of which, similarly, more who agreed were female (61.4% compared to 41.3%), and those with children were more likely to agree than those without (Table 2). As before, we found that more people agreed with the statement in the regions of Quebec, Ontario, and Atlantic Canada. Another finding which came from this question, perhaps unexpectedly (although an argument could be made both ways), is that those with a household income of less than CAD 50,000 per year were more likely to agree than those with a household income over CAD 50,000.

Consumers, for the most part, feel they pay a fair price for milk in Canada, and that drinking milk produced in Canada is more important than having milk at lower prices (57.2% agreed with the statement (Table 3): “*The price of milk and other dairy products is fair in Canada.”* A total of 55.8% disagreed with the statement: *“I believe lower prices for consumers is more important than drinking Canadian milk”*) (Table 4). Finally, on this note, Canadians also agreed (66.3% of the time) with the statement: *“If dairy products were more expensive, I would still purchase them if it means that we can keep dairy farms in Canada.*”

Again, a recurrent theme in this study, women were more likely to agree than men for both statements. Another recurring theme: when divided by region, we find again that individuals from Quebec, Ontario, and the Atlantic region were slightly more likely to agree with the statements.

Consumers also raised other concerns, making their preferences clear regarding how the dairy industry should be managed in Canada. Canadians identified that they are concerned with the environmental impact of the dairy industry, the welfare of animals in the dairy industry, the quantity and quality of available dairy products, and showed overwhelming support for protecting rural Canada. Turning first to the environmental impacts of the dairy industry, we determined that most Canadians did not agree with the statement: *“I believe dairy farming is good for the environment”* (39% agreed) (Table 5). In fact, Canadians say they would be willing to pay more for dairy if it had less of an impact on the environment (68.5% agreed with the statement: *“I am willing to pay more for food which is sustainably sourced and has less of an impact on the environment*”). Interestingly, when we analyze the data by education, we find that those with higher levels of education (an undergraduate degree or higher) are less likely to agree that dairying is good for the environment than those with less education. We also found that those who feel that dairying has a negative impact on the environment are more likely to be women, and, again, are more likely to be of a younger generation.

When considering the welfare of animals, Canadians made it clear: Canadians do not feel the dairy industry protects animal welfare in Canada (especially younger generations), and they would be willing to pay more to ensure it does, as 73.7% agreed with the statement: *“I am willing to pay more for food which ensures animals are treated humanely”* (Table 6). They also believe that small farms treat animals more humanely than large farms, with 55% disagreeing with the statement: *“I believe large dairy farms treat animals more humanely than small farms.”* One respondent even proclaimed:

“Honestly, milk is a largely inhumane industry more interested in selling the white cow juice and making a buck than focusing their acquired resources on other more sustainable nutritional options.”

This sentiment, that animals are treated unfairly in the dairy industry in Canada also corresponds to level of education, with a higher level of education correlating with a low percentage of agreement. Interestingly, only 46.9% of people living in urban cores agreed with the statement, while 66.3% of those in rural areas agreed.

Finally, consumers also asked for a greater variety of products to choose from, and greater support for rural Canada. A total of 61.8% agreed with the statement: *“I would prefer a greater variety of dairy products available to me, such as artisan cheeses and yogurt.”* These numbers were, as expected, higher amongst individuals whose household income was above CAD 50,000 per year.

## 4. Policy Implications

The Canadian dairy industry is currently facing challenging times. Recognizing the difficulties of Canadian dairy farmers and the implications of current dairy policy, there are 5 overarching themes that are inspired by the opinions of Canadians and supported by fundamental underpinnings found through the AAL national survey. They are as follows:

1. Maintain dairy farming, and processing, in all regions.
2. Allow dairy farming to become more financially appealing.
3. Make our domestic production capacity more competitive.
4. Adopt a value chain-focused approach to a reform.
5. Develop a strategy which focuses on innovation and growth, both domestically and internationally.

These themes drawn from the study suggest that the actions of government do not fully align with the goals of Canadians regarding the supply management system and the Canadian dairy industry. The only exception is that the Liberal government is currently providing compensation through direct subsidies to dairy farmers for losses from trade negotiations, thus making the industry more financial appealing. This said, the compensation is being provided without a clear strategy, is creating a precedent, and will overcapitalize the industry. It is evident that the GoC did not take the considerations of the public when making current policies, thus our hypothesis was not supported. Overall, it is unclear how our current path can achieve any of these goals.

Policy should be evaluated based on meeting the wants of the Canadian public, though the current regime lacks this and instead chooses to support the wants of lobbyists organizations like DFC. Declining fluid milk sales and increasing international imports are not the only issues facing dairy farmers. The support of the dairy industry in younger generations is fading. While effort on the part of DFC along with the aforementioned direct subsidies for dairy farmers from the Liberal government may save the livelihood of Canada’s dairy farmers in the short term, certainly, looking forward, changes to the current system are needed.

The Canadian dairy sector will need to think differently about its future. Other countries where supply management was eliminated have had to manage different economic realities, but supply management here in Canada has severe limitations. Deciding what needs to be changed regarding dairy supply management in Canada is not an easy task, but the industry is under pressure. Approaches like how the current milk pricing quota, as set by the CDC in cooperation with the Provincial Milk Marketing Boards as voting members of the CMSMC, is merely revealed is not enough. Additionally, most of our dairy farms and processors are in Central Canada (74% of all dairy farms are in Ontario and Quebec which have only 61% of the total population), leaving many regions underserved. We must reevaluate how that price is determined altogether, potentially using a value-chain focus approach and incentivizing the formation of cooperatives between small farms in each region. We could set a higher farm-gate price for dairy to recognize the increased input costs of production for smaller producers. If we are to maintain a system, it needs to foster innovation across the country, while keeping in mind some of the market challenges agriculture faces. This concept of supporting rural through the form of promoting and encouraging rural dairy farms is backed by public opinion, which contrasts with the centralization of dairy farms that have been rising. This would lead to increased jobs for rural Canadians, which is favored by most Canadians, and smaller dairy farms, which 55% consider to be more humane for dairy cows than larger ones. Consumers are increasingly concerned about the environment and animal welfare. Given that the sector is focused on supply-side economics, it is ill-prepared to face these concerns expressed by consumers, especially the younger generations.

Policy recommendations must rest on the foundation that the country needs a strong nationwide dairy sector, and that Canadians want to eat Canadian-produced commodities. Canadians are even willing to pay more for dairy products which would allow for dairy farming to be more financially appealing. A leading factor of industrial centralization is a response to rising costs of production, this is true regarding the Canadian dairy industry. Considerations must be made knowing that 66% of Canadians are willing to pay in excess for dairy products if they are produced in Canada and that 50% of Canadians support direct subsidies to these Canadian dairy farmers. Financial stability for Canadian dairy farmers is a relevant factor when determining new dairy policy and is clearly supported by the public.

Overall, innovation and dairy processing are key for the sector’s growth and future, and that regional proportionality is critical for all Canadians. Maintaining domestic production capacity is vital to the success of the sector and to our nation’s food security, though these themes are potentially misguided since the large majority of Canadians do not fully understand, or feel that they understand, the supply management system. What needs to be determined is the optimal number of dairy production operations needed in Canada, and no one has the answer.

Some limitations of the AAL national survey resulted from the use of Likert Scale scoring and the resulting biases. Notably, this survey may have been susceptible to false reporting due to acquiescence bias and extreme/neutral responding bias, due to the agree/disagree type questions. Further biases may have been the result of sampling and non-response bias. Additional errors in the result may have been due to self-report bias because participants could have overestimated their knowledge, been dishonest, or not understand the questions asked. Moreover, participants may have felt uncomfortable answering truthfully about their knowledge of supply management and the Canadian dairy industry, feeling inadequate, thus causing a desirability bias.

## 5. Conclusions

Though many experts in the field have pushed for Canada to follow Australia’s lead and eliminate supply management, in turn eliminating government compensation and subsidies, Canadian care about where their food comes from and would prefer to support food grown in Canada. The dairy sector is an important component to the country’s rural economy. This is clear with their support of the financial stability of Canadian dairy farmers. The fact that the Canadian public now financially supports the dairy sector changes everything. It can be argued now that the social contract between the sector and Canadians has been renewed and stakes are different. Unlike other countries, milk and butterfat can be considered as public goods. Canadians protect, compensate and support the industry by virtue of highly protectionist policies. For that moral contact to be successful, many things need to change.

The sector needs to be made more competitive, accountable, and transparent. Without a strategy, the compensation program provided by the federal government will make things worse for the dairy sector and the farmers themselves. It will overcapitalize the market, without fostering competitiveness. Understanding how food policy is being developed should therefore be of crucial concern to Canadians. While the issue may have become political, the fact remains, if supply management continues as it has, we will continue to see losses of Canada’s domestic market to Free Trade Agreements and a lack of innovation in dairy processing, thus we will continue to have regional amalgamation of farms in the form of economies of scale, in effect harming smaller farms, and further separating reality from the wants of Canadians.

## Figures and Tables

**Table 1:** I believe supply management is good for the Canadian economy.

In which Region Do You Currently Reside?						
	Total	Atlantic Canada	British Columbia	Ontario	Prairies	Quebec
I believe supply management is good for the Canadian economy.						
Strongly agree	21.6%	26.0%	17.5%	24.4%	17.5%	20.7%
Somewhat agree	38.7%	34.2%	38.5%	39.2%	31.6%	45.0%
Somewhat disagree	9.5%	6.8%	12.6%	10.9%	11.9%	4.5%
Strongly disagree	5.4%	6.8%	4.2%	6.2%	7.3%	2.9%
Overall Stat Test of Percentages	0.04829					

**Table 2:** I believe it is important to support Canadian dairy farms by providing direct subsidies to them.

	Total	Female	Male
Strongly agree	20.7%	27.9%	13.2%
Somewhat agree	30.9%	33.5%	28.1%
Somewhat disagree	13.6%	7.8%	19.6%
Strongly disagree	10.3%	5.0%	15.8%
Overall Stat Test of Percentages	0.00000		

**Table 3:** The price of milk and other dairy products is fair in Canada.

	Total	Female	Male
Strongly agree	17.5%	20.8%	13.8%
Somewhat agree	39.7%	44.7%	34.5%
Somewhat disagree	14.9%	11.1%	18.8%
Strongly disagree	9.4%	4.2%	14.8%
Overall Stat Test of Percentages	0.00000		

**Table 4:** I believe lower prices for consumers is more important than drinking Canadian milk.

	Total	Female	Male
Strongly agree	7.8%	4.8%	11.0%
Somewhat agree	15.7%	12.2%	19.4%
Somewhat disagree	31.3%	31.4%	31.3%
Strongly disagree	24.5%	30.8%	17.8%
Overall Stat Test of Percentages	0.00000		

**Table 5:** I believe dairy farming is good for the environment.

	Total	Before 1946	From 1946 to 1964	From 1965 to 1979	From 1980 to 1994	After 1994
Strongly agree	17.2%	38.1%	18.9%	16.1%	11.9%	16.3%
Somewhat agree	22.0%	23.8%	22.8%	26.3%	17.7%	18.8%
Somewhat disagree	20.7%	14.3%	14.4%	19.2%	28.0%	30.0%
Strongly disagree	8.7%	0.0%	5.4%	7.5%	13.7%	15.0%
Overall Stat Test of Percentages	0.00000					

**Table 6:** I believe the dairy industry treats cows and other animals humanely in Canada.

	Total	Before 1946	From 1946 to 1964	From 1965 to 1979	From 1980 to 1994	After 1994
Strongly agree	20.5%	31.7%	20.4%	20.8%	18.1%	20.0%
Somewhat agree	35.3%	46.0%	39.9%	34.5%	30.4%	27.5%
Somewhat disagree	11.5%	1.6%	9.3%	11.8%	14.3%	17.5%
Strongly disagree	6.9%	0.0%	6.0%	4.3%	10.2%	12.5%
Overall Stat Test of Percentages	0.00013					

## Data Availability

As per MDPI Research Data Policies.
